# New insights into the ontogeny of human vegetable consumption: From developmental brain and cognitive changes to behavior

**DOI:** 10.1016/j.dcn.2020.100830

**Published:** 2020-07-25

**Authors:** Paloma Rohlfs Domínguez

**Affiliations:** Department of Psychology and Anthropology, University of Extremadura, Faculty of Nursing and Occupational Therapy and Faculty of Teaching Training, Avenida de la Universidad, s/n 10004, Cáceres Spain

**Keywords:** Brain, Cognition, Changes, Children, Ontogeny, Vegetable consumption

## Abstract

Relatively little is known about how mental development during childhood parallels brain maturation, and how these processes may have an impact on changes in eating behavior: in particular in vegetable consumption. This review aims to bridge this research gap by integrating both recent findings from the study on brain maturation with recent results from research on cognitive development. Developmental human neuroscientific research in the field of the sensory systems and on the relationship between children’s cognitive development and vegetable consumption serve as benchmarks. We have identified brain maturation and mental growth patterns that may affect child vegetable consumption and conclude that both of these developmental patterns partially match with the Piagetian theory of development. Additionally, we conclude that a series of potential modulating factors, such as learning-related experiences, may lead to fluctuations in the course of those particular developmental patterns, and thus vegetable consumption patterns. Therefore, we propose a theoretical predictive model of child vegetable consumption in which the nature of the relationship between its correlational and/or causal components should be studied in the future by adopting an integral research perspective of the three targeted study levels: brain, cognition and behavior.

## Highlights

• There is research gap regarding how mental growth and brain maturation may impact on vegetable consumption.
• We have identified particular brain maturation and mental growth patterns that may affect child vegetable consumption.
• Both of these developmental patterns partially match with the Piagetian theory of development.
• We have identified a series of potential modulating factors.
• The 3–4 and 4−5 age ranges might potential sensitive periods for acquisition of brand knowledge of foods and health-related abstract concepts.

## 1 Introduction

Most humans make their food choices, which are defined as «the selection of foods for consumption, which result from the competing, reinforcing and interacting influences of a variety of factors» (Marty et al., 2018, p. 466) on the basis of its flavor (Beauchamp and Mennella, 2011; Havermans, 2016). This tendency is even more pronounced among children, who usually “eat what they like” (Birch, 1999; Burguess-Champoux et al., 2006) and naturally tend to like sweet tastes and reject bitter tastes. Indeed, children commonly reject vegetables: especially cruciferous (green) vegetables, such as watercress and kale. The preference is particularly evident in ‘taster children’ who are children with a high genetic predisposition to taste the bitter taste of a given food (see Keller and Adise (2016) and Rohlfs-Domínguez (2017) for reviews).

However, a diet rich in vegetables helps prevent diseases, such as childhood obesity (Anderson and Butcher, 2006; Leahy et al., 2008; Aranceta-Batrina and Pérez-Rodrigo, 2016) and cancer during adulthood (Van Duyn and Pivonka, 2000). Furthermore, the patterns of food liking and consumption that have been learned early in life may also last at least until young adulthood (Nicklaus et al., 2005; Pearson et al., 2009: Wadhera et al., 2015b) so are therefore associated with the state of health in adulthood (Qi and Niu, 2015; Yzydorczyk et al., 2015; Rolland-Cachera et al., 2016). This prior research allows the hypothesis that the flavors that lead to food choices made during childhood have implications for the individuals’ future state of health.

Flavor is a property of foods and drinks that is mainly generated by the integrated processing of two main kinds of sensory-chemical information: tastes and odorants, which occurs in both the gustatory and olfactory sensory systems respectively (Beauchamp and Mennella, 1998; Simpson and Sweazey, 2006). The gustatory and olfactory functions are present at birth (Rosenstein and Oster, 1988; Faas et al., 2000) and their development continues postnatally (Mennella et al., 2005). Humans may perceive five different taste qualities: sweet, salty, bitter, sour and umami (Yoshida et al., 2006; Medler, 2008). The tastants commonly associated with these tastes are sucrose (sweet), sodium chloride (salty), citric, malic or tartaric acid (sour) quinine or caffeine (bitter) and monosodium glutamate (MSG) (umami) (Heymann and Ebeler, 2017). The number of odors that can be discriminated by humans has been estimated to be around half a million (Kringelbach, 2007). Although the gustatory and olfactory systems are anatomically separated from each other, flavor is perceived as a single perceptual unit (Beauchamp and Mennella, 2011). This occurs because of the integrative work of our nervous system: in particular, our central nervous system (CNS).

Use of neuroimaging techniques has become indispensable in basic and applied research for obtaining information about the structures and functions of the human brain. Advantages of these techniques are the high temporal and spatial resolution, which overcomes spatial limitations of the electrophysiological techniques; their non-invasive quality, which permits measurement of neural activity without the necessity of surgically placing electrodes in the brain; and the possibility of conducting these measurements *in vivo*. Examples of these neuroimaging techniques are magnetic resonance imaging (MRI), functional MRI (fMRI), perfusion imaging (PI) (see Toccio et al., 2015, for a review) and functional near-infrared spectroscopy (fNIRS). The aim of using these techniques in experimental contexts, from a developmental perspective, is to identify or find, within the targeted systems, changes in brain activation patterns that are associated with age-related changes. The underlying goal is, therefore, to know how those systems develop during maturation. Changes in brain activation patterns are expected to be observed as a function of age, given the plastic nature of the CNS: neural plasticity. According to Lourenco and Casey (2013), we can define neural plasticity as the ability of the CNS to adapt its functioning and anatomy according to experiences of environmental changes and demands, which are indispensable to promote individuals’ survival.

Behavioral research conducted with children has shown that early repeated exposure to flavors, including the flavors of vegetables, may induce increases of consumption of the foods with those flavors (see Rohlfs-Domínguez, 2014a, for a review). Tasting the flavors of these foods at early stages of development: specifically at the prenatal, breastfeeding, and weaning stages, along with early childhood from 2 to 6 years of age, causes children to learn to like those foods and vegetables (see Rohlfs-Domínguez, 2014a; Wadhera et al. 2015; Rohlfs-Domínguez, 2018; Spahn et al., 2019; Spill et al., 2019 for reviews). Moreover, there is evidence pointing to the existence of sensitive periods of gustatory and olfactory learning that shows high correlations between the acquired food preferences during early childhood and the food preferences in posterior life phases, such as late childhood, adolescence and young adulthood (see Rohlfs-Domínguez, 2014b, p. 32 for a review). Sensitive periods are «restricted periods of time in development, during which there is […] an extreme neural sensitivity to the storage of experience-driven sensory and probably conceptual information. […] The main effects of exposure to stimuli [*i.e.* particular foods] during a sensitive period are the lasting or everlasting changes in individual’s behavior [*i.e.* preferences for, or consumption of, those foods…]» (Rohlfs-Domínguez, 2011b, p. 909). The development of «higher functions, such as learning to read, […] learning a second language, and learning to understand abstract concepts, such as the healthiness of a food, including vegetables […], can [also] occur if there is exposure to specific kinds of experiences. A child, who has never been read to, or never seen a book, is not going to develop reading skills» (Bailey, 2002, p. 287).

Most children base their food choices on affective appraisals of the sensory attributes of foods, such as flavor. Some children, and many adults, make their choices on the basis of more cognitive attributes, such as the healthiness or nutritive content of a given food (Pérez-Rodrigo et al., 2003; Navarro-Allende et al., 2008), which requires an understanding of abstract concepts, such as the healthiness or nutritive content (Contento, 1981). The differences in the kind of food attributes that are used to make food choices may be due to, or correlate with, differences in the degree of cognitive development that has been achieved, which enables understanding and manipulation of such abstract concepts. According to McNeal (2007), cognitive development refers to changes in knowledge, and in the use of knowledge, which occur with increasing age (McNeal, 2007).

Piagetian theory of child cognitive development is the most used theory when studying different aspects of human cognitive development. This theory proposes that each child develops across a series of different stages of mental growth. According to Pulaski (1997), these development stages are: 1. Sensory-motor (0–2 years) in which infants’ behavior is based on innate reflexes, such as suction and pure sensory-motor experiences: There is no intentional mental activity. 2. Preoperational (2–7 years) in which behavior is based on pre-logical reasoning and symbolic representation: Learning is based on trial and error. 3. Concrete operational (7–11/12 years) in which logical reasoning and ability to classify objects into categories is used: Intellectual development moves sharply upwards. 4. Formal operational (11/12 years onwards), in which the person develops the ability to generate hypotheses, to use deductive, inductive and abstract thinking and to handle large amounts of data. Children do not use causal reasoning during the two first stages but begin to use it in the third stage (Contento, 1981). During the preoperational period, language develops but the «child tends to repeat words and sentences without comprehension because he or she does not yet have access to the basic cognitive structures for understanding them. […]» (Contento, 1981, p. 86). Piaget concluded that a series of factors affect children’s cognitive development: maturation, that is, biological development and physical growth; experience, which refers to the formation of relationships with the stimuli, such as objects in the surrounding environment; and social transmission of information, which refers to any knowledge that is learned from related persons (Baskale et al., 2009).

The aim of this contribution is to identify the sequence of brain, cognitive and behavioral changes that have to be undergone by children for them to consume vegetables or even to increase this consumption. To operationalize this aim, we consider the following questions: how do relevant sensory systems develop postnatally as age increases? How is this development reflected in brain activation patterns, which are measured through neuroimaging techniques? How does this development relate to brain development and mental growth during the potential sensitive period of learning to understand abstract concepts, such as healthy foods? How might this process impact children’s vegetable consumption?

In responding to these questions, we first discuss a series of different neuroimaging studies that have been carried out for the analysis of the postnatal development of chemical senses and the product of its integration: expressed as flavor perception. Due to the scarcity of studies that have used fMRI in children, and for reasons of brevity, the focus of this section is limited to fMRI studies. We then address results of research work that has focused on the relationship between children’s cognitive development level, their ability to categorize foods as healthy or unhealthy, and their ability to understand the healthiness of vegetables, which is an abstract concept that might cause positive changes, such as increases in child vegetable consumption. In a third section, we address parallels between brain and cognitive development as well as the question of how both of these processes might contribute to child vegetable consumption. Finally, we conclude with recommendations on how this contribution might help in the design of strategies for increasing children’s vegetable consumption, by considering their brain maturation and cognitive development level.

## 2 Postnatal development of sensory systems activated by food stimuli: evidences from developmental neuroimaging studies

The question to be addressed here is whether there are identifiable age-related changes in postnatal brain activation patterns in response to food stimuli. Identification of these brain activation patterns may help provide information about the existence of specific patterns of postnatal brain development that affect gustatory, olfactory, and flavor processing. Such patterns might be developed in parallel with progress in cognitive development and are likely to be associated with rates of vegetable consumption. Although English et al. (2015) have developed a protocol for using fMRI with 7-to-10-year-old (yo) children, studies that have used fMRI in children are scarce, which is probably due to difficulties with conducting fMRI in child populations (see Keller and Bruce, 2018, p. 214).

**Fig. 1:**
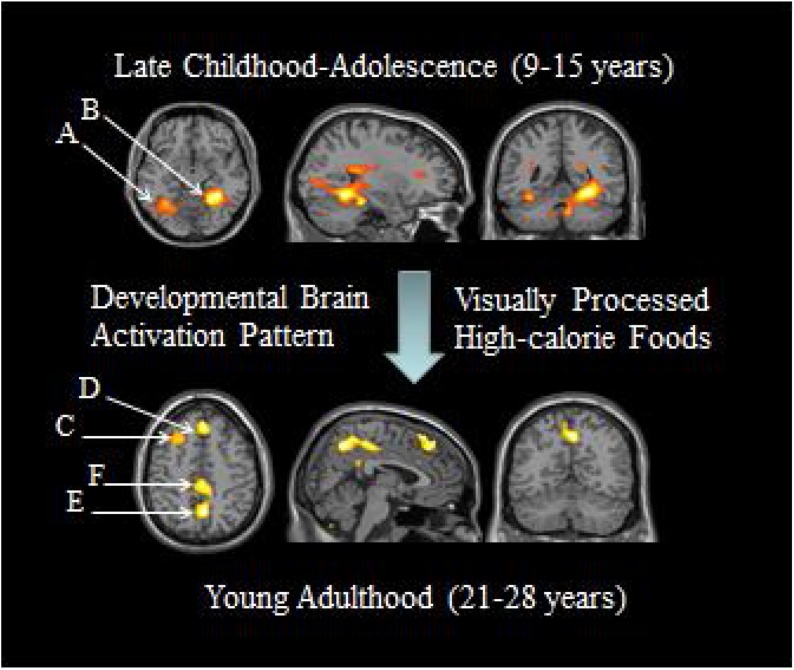
The younger group shows greater activation than the older group in the fusiform gyrus (A) and the precuneus (B) whereas young adults show greater activation than the younger sample in the medial prefrontal (C), dorsolateral prefrontal (D), precuneus (E) and posterior cingulate (F) regions. Adapted from Killgore and Yurgelun-Todd (2005) (Image courtesy by Dr. W.D.S. Killgore).

**Fig. 2:**
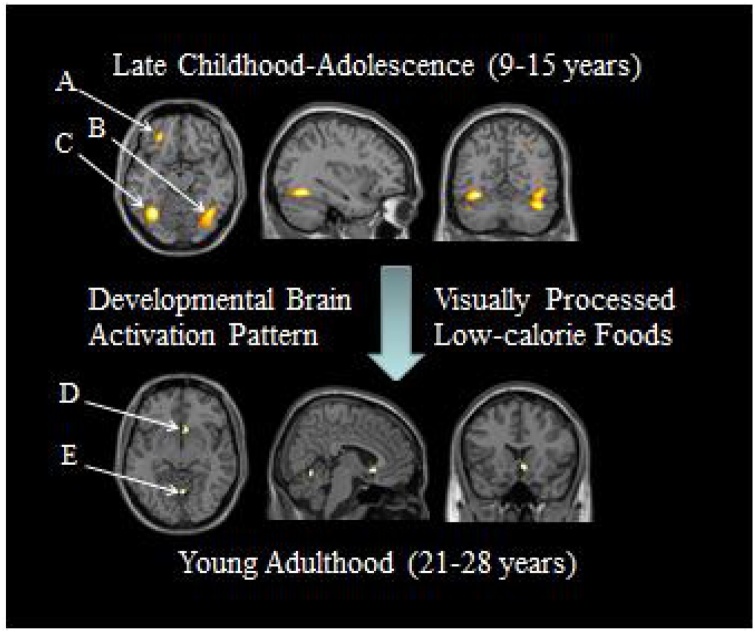
The younger group shows greater activation than the older group in the OFC (A), fusiform gyrus (B) and the cerebellum (C), whereas the older subject sample shows greater activation than the younger sample in the olfactory cortex (D) and the vermis of cerebellum (E). Adapted from Killgore and Yurgelun-Todd (2005) (Image courtesy by Dr. W.D.S. Killgore).

**Fig. 3:**
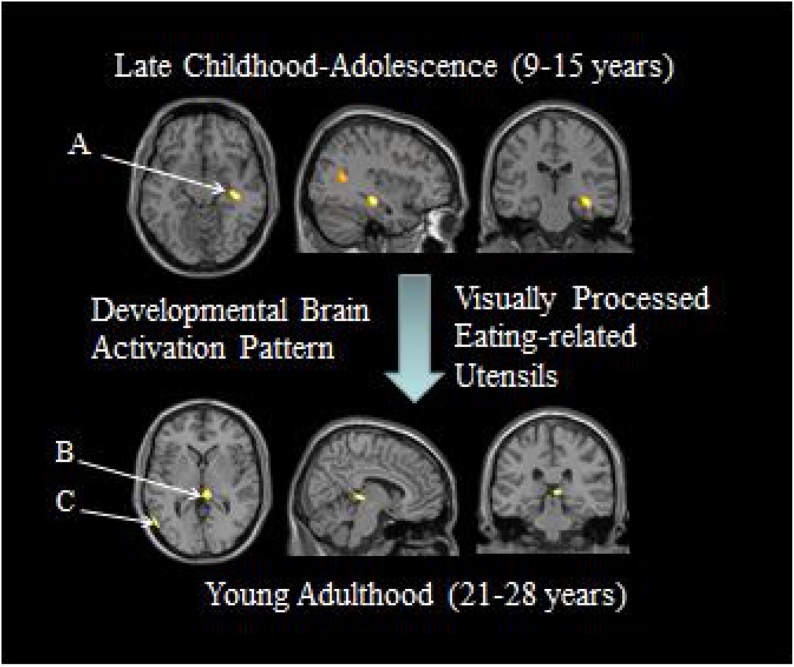
Adolescents show greater activation than the young adults in the right hippocampus (A), and these participants show greater activation than the younger subjects in the right posterior thalamus (B) and the left middle temporal gyrus (C). Adapted from Killgore and Yurgelun-Todd (2005) (Image courtesy by Dr. W.D.S. Killgore).

A review by Rohlfs-Domínguez (2011a) pointed out a line of research that has focused on examination of neural activations in response to visually presented food-related images by means of fMRI. This review outlined the study by Killgore and Yurgelun-Todd (2005) who found age-related differences in brain activation patterns in response to high-calorie foods, low-calorie foods and eating utensils between a sample of female subjects aged from 9 and 15 years and a sample of young adults aged from 21 and 28 years. On the basis of these results, Rohlfs-Domínguez (2011a) concluded that: humans process food-related visual, gustatory, olfactory, hedonic, and cognitive information simultaneously. When presented with images of foods or food-related utensils, the ability to process all this information increases with increasing age, or maturation, which is reflected by an increase of the BOLD signal. Furthermore, Rohlfs-Domínguez (2011a) proposed that high-order information processing, in addition to sensory features (low-order sensory processing) of those stimuli, is engaged as subjects mature (See Fig. 1, Fig. 2, Fig. 3).

**Fig. 4:**
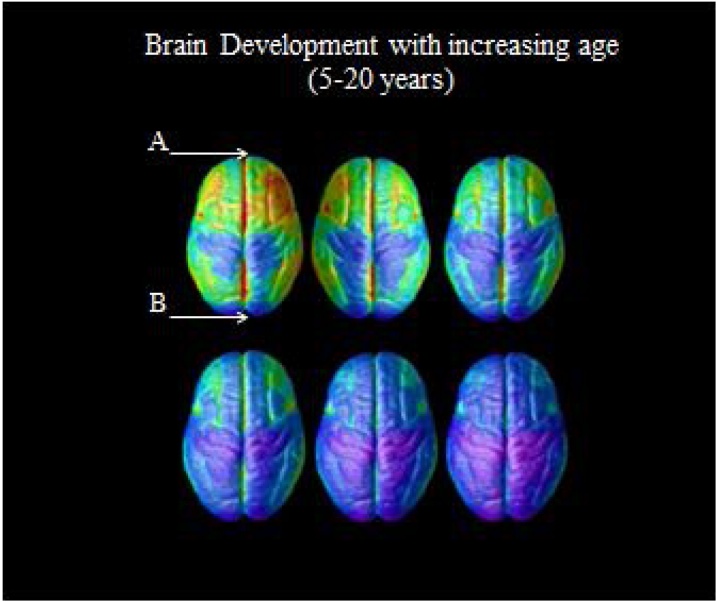
More rostral brain areas (A) mature later than the more caudal brain regions (B). Adapted from Gogtay et al. (2004) with permission (Copyright (2004) National Academy of Sciences).

That progressive shift from low-order to high-level neural processing of visually presented, food-related stimuli agrees with previous evidence from anatomical and functional brain mapping in healthy individuals. Under disease-free conditions, more rostral cortical brain regions mature, and thus become completely functional in later stages of development than caudal cortical structures do. The rostral cortical brain regions include the prefrontal cortex, which is involved in high-order processing, such as executive functions. The caudal cortical structures, which include the occipital cortex, are involved in low-order processing, such as visual processing. This development pattern has been called progressive “back-to-front” cortical brain development (Gogtay et al., 2004; Zalesky et al., 2015) (See Fig. 4).

Although children younger than 6-yo were not enrolled in this study, we may hypothesize less neural activity in high-order processing brain areas in comparison to low-order processing brain areas in response to visually presented foods and food-related stimuli. Additionally, we may also conclude that the targeted progressive shift in neural processing dovetails with the behavioral changes that Piaget observed during childhood development. «According to the Piaget classification of childhood developmental stages [children first interact with the surrounding stimuli] […] through movement and [senses, such as seeing] -“sensorimotor intelligence’’-. Subsequently, the child learns to integrate visuospatial information into the concrete object representations -“preoperational intelligence’’-. These new visuospatial abilities are mediated primarily by posterior parietal cortex and occipital cortex. Only later does the older child build a logic of concrete operations -“operational intelligence’’-, which becomes a rational hypothetico-deductive system -“formal operations’’- during adolescence. These last two stages of child development are heavily dependent on frontal lobe function especially prefrontal cortex. [Experimental research has shown] that children often fail to inhibit perceptual reasoning biases in favor of deductive logic for problem solving» (Mukherjee and McKinstry, 2006, p. 27).

Van Meer et al. (2016) examined and compared neural responses to pictures of healthy and unhealthy foods among 10-to-12-yo children/adolescents and their parents aged 32–52 years. The younger group showed greater activation to unhealthy, relative to healthy, foods in the inferior frontal gyrus and middle frontal gyrus, which are implicated in cognitive control as well as in the hippocampus, which is implicated in reward processing and memory. Adults showed greater activation to unhealthy, relative to healthy foods, only in the middle occipital gyrus and right calcarine sulcus, which are visual processing regions. In addition, when viewing unhealthy foods, the younger group showed a greater activation than adults in the left precentral gyrus, which is implicated in motor coordination and planning (van Meer et al., 2016). The authors of this study suggest that the greater activation in this region may reflect motor planning and motivation for ingestion (van Meer et al., 2016), which suggests that the older children/adolescents and probably young children show a greater readiness to consume those unhealthy foods than adults do.

The meta-analysis by Van Meer et al. (2015) identified common regions that responded to visual food stimuli in both adults (age range = 22−44-yo) and children (age range = 10−18-yo): the bilateral fusiform gyrus, the left lateral orbitofrontal cortex, and the right superior parietal lobule. However, they also outline some differences. Indeed, activation in the lateral orbitofrontal cortex was more frequent in children than in the adults, while the opposite pattern was identified for activation in the fusiform gyrus. Furthermore, children activated the prefrontal cognitive control regions to a lesser extent than adults. The authors concluded that adults process food-related visual stimuli using a wider, and thus more complex, system of neural circuits than older children and adolescents. We hypothesize that young children would also show less activation in prefrontal cognitive brain regions in comparison to adults.

The study by Holsen et al. (2005), in which the neural response to visual food stimuli was examined and compared among 10-to-17-yo participants during fasted and fed states, however, yielded brain activations in similar brain regions, such as the orbitofrontal cortex, amygdala and insula. As no age effect was found, Holsen et al. (2005) concluded that the normal, disease-free, appetitive-related neural circuitry is present in childhood and remains constant from late childhood to adolescence. This study was conducted with older children and adolescents rather than young children, so does not allow conclusions as to whether the stability of that neural circuitry changes from young childhood to late childhood and, by extension, from adolescence to adulthood.

Rohlfs-Domínguez (2011a) also identified an asymmetric hedonic-related brain activation pattern in both newborns and young adults in response to olfactory stimuli, in which pleasant odors show greater left than right brain activation in the OFC, and the opposite brain for unpleasant odorants. Therefore, this cortical brain lateralization in response to olfactory stimulation is already present at birth and remains constant across development: at least from birth towards young adulthood (Rohlfs-Domínguez, 2011a). A series of studies have shown that tasty foods and energy-rich foods activate brain regions involved in emotional processing: hedonic value, and rewards: food rewards, such as the anterior cingulate gyrus, the caudate and *substantia nigra* along with motivation: hunger, in 7-to-10-yo children (English et al., 2016; Fearnbach et al., 2016; English et al., 2017). These findings suggest that neural processing of food reward and the hedonic value of food is present from late childhood onwards. Since neural processing of the hedonic value of chemical stimuli is already present at birth, we may hypothesize that neural processing of food reward is already present at young childhood.

A person’s ability to reason about the appropriateness of a particular food on the basis of its nutritive content and health-related effects on the body requires sharp comprehension of the effects that this food may have on the body as well as the implications of supplying the body with nutrients (Contento, 1981). Evaluation of these abstract concepts implies use of logical and relational thinking. However, neuroimaging studies that have examined the development of neural circuits that underlie the development of understanding and manipulation of abstract concepts, such as healthy foods in an *ad hoc* way are surprisingly lacking. Indeed, according to Binder and Fernandino (2015), «much of the […] neuroimaging research has so far focused mainly on concrete objects, action, and emotion knowledge; more study is needed concerning […] abstract concepts» (Binder and Fernandino, 2015, p. 445). One exception is a recent review by Dumontheil (2014) who identified a prolonged development of these cognitive functions, which are typically associated with the rostrolateral prefrontal cortex (RLPFC), from structural and functional neuroimaging studies that have confirmed a prolonged development of this brain area during adolescence.

## 3 Children’s stage of cognitive development and vegetable consumption

Several studies have examined the impact of age-related developmental cognitive changes in the use or understanding of abstract concepts, such as vegetables and other healthy foods, on children’s food choices. These studies have evaluated whether the previously described shift in neural processing, and thus temporal sequence of changes, is reflected in children’s cognitive development and thus food choices. Most of these studies are based on Piaget’s theory of child development.

A series of research work broadly shows that young children who are supposed to be located at the preoperational stage of cognitive development do not use, or do not understand, concepts such as healthy foods or the healthiness of a given food or nutrients. Furthermore, young children are less able to categorize foods into healthy and unhealthy categories than older children, who are located at the concrete operational stage of cognitive development. An overview of these studies is shown in Table 1.

**Table 1:** First set of studies reviewed.

Citation	Sample (n participants)	Children’s age	Method	Children’s previous cognitive classification
Contento (1981)	n = 34	5-to-6-yo	Quantitative; Interview	Yes
Bahn (1989)	n = 52	4-to-5-yo	Quantitative; Interview and Judgment tasks.	No
Fallon et al. (1984)	n = 29	3.5-to-12-yo	Quantitative; Rating scales; Interview	No
Nu et al. (1996)	n = 222	10-to-20-yo	Quantitative; Questionnaire	No
Zeinstra et al. (2000)	n = 28	4-to-12-yo	Qualitative; Interview, Focus group discussions, Game tasks and Food tastings	No
Frerichs et al. (2016)	n = 38	8-to-13-yo	Quantitative and Qualitative; Interview and focus groups	No

Contento (1981) found that preoperational stage children from 5-to-11-yo do not differentiate between foods and snacks, which are considered as unhealthy foods because of their high sugar and fat content (Warren et al., 2008), while concrete operational stage children make this differentiation. She also found that preoperational stage children have more difficulties to understand food- health-, nutrition- and digestion-related questions than concrete operational children. She concluded that differences in children’s food choices may lie in their cognitive differences regarding such abstract questions. In this study, the children had previously been classified into preoperational and concrete operational stages on the basis of standard Piagetian tasks. As a result, one 6-yo child, one 8-yo child and eight out the ten 5-yo children were classified as preoperational stage children whereas two 5.5-yo and twenty-two 6-to7-yo children were classified as concrete operational stage children. In a subsequent study, however, it was found that 5-to-11-yo children do differentiate between foods and snacks, but understanding of nutrients is in general poor, this understanding increasing with cognitive development (Michela and Contento, 1984).

Bahn (1989) concluded that 4-to-5-yo preoperational and 8-to-9-yo concrete operational stage children base their preferences for beverages on perceptual attributes, such as sugar content; affective appraisals, such as liking the taste; and cognitive attributes, such as healthiness. In this study, the children were also previously classified into preoperational and concrete operational stages on the basis of standard Piagetian tasks. Similarly, Fallon et al. (1984) observed, in 3.5-to-12-yo children, that the criterion used for rejecting a food is age-dependent. The youngest children based their food rejections on the taste; older children considered the potential harmful post-ingestive consequences of eating a given food; and the oldest children considered the contamination by physical chemistry of particular liquid solutions. A posterior study by Nu et al. (1996) observed, in a sample of 10-to-20-yo participants, that pre-pubescent adolescents rejected many foods that they had previously liked, such as fish, offal, seafood, and spinach, which the authors called ‘negative changes’. The authors also noticed that, after puberty, individuals begin to like some particular foods that they did not like in previous life stages, such as tomatoes, spinach, carrots, and cabbage, which they called ‘positive changes’. The authors attribute the negative changes to physiological reasons, such as sickness, and the positive changes to cognitive factors, such as an increased ability to understand relationships between food consumption and health at puberty: an ability that emerges once individuals have reached sufficient cognitive development. Zeinstra et al. (2007) deduced that 4-to5-yo children can neither correctly categorize edibles into healthy and not healthy products nor argue the underlying reasons for the healthiness of certain “healthy” foods, but are able to complete both of these tasks in later childhood: from the age of 7-yo.

Frerichs et al. (2016) aimed to explore how children understand food and healthy eating by studying 8-to-13-yo children. They found that children do not use such causal relationships to determine healthiness, but instead use a heuristic based on major food groups, such as fruits and vegetables, while not strongly connecting health values with foods they liked. Furthermore, they found that likeability in children is primarily shaped by sensory attributes of foods, such as taste, texture, and visual appeal, and that they associate liked edibles with positive home- and family-related experiences. Here again, lack of understanding of food-related abstract concepts may explain, at least partially, why children, in general, and young children in particular, do not consume sufficient healthy but (in the children’s opinion) bad-tasting, vegetables. A further series of studies, however, has found that the ability of young children to use, or to understand, abstract concepts and categorizations may be more limited than in their older counterparts rather than absent, which implies that young children may require additional support for understanding such abstract concepts (see Table 2).

**Table 2:** Second set of studies reviewed.

Citation	Sample (n participants)	Children’s age	Method	Children’s previous cognitive classification
Lytle et al. (1997)	n = 14	4-to-12-yo	Qualitative; Interview and Educational focus groups.	No
Nguyen (2007)	n = 48 and n = 16	3-to-7-yo and 18-to-20-yo	Quantitative; Categorization task.	No
Tatlow-Golden et al. (2013)	n = 172	3-to-5-yo	Quantitative; Questionnaire, Interview and Storybook.	No
Gripshover and Markman (2013)	n = 59 and their parents.	4-to-5-yo	Quantitative; Focus groups, Interview, Observation, Questionnaire and Storybook.	No
Varela and Salvador (2014)	n = 148	5-to-9-yo	Quantitative; Interview, Rating scale and Sorting task.	No
Dial and Musher-Eizenman (2019)	n = 40	4-to-6-yo	Quantitative and Qualitative; Categorization task and Interview.	No

Lytle et al. (1997) studied how 4-to-12-yo children understand and use nutrition messages. They noticed that 4-to-10-yo children show more difficulties in interpreting abstract terms, such as variety and healthy weight, than 10-to-12-yo children. Furthermore, they reported that it is difficult for all children to name high-fat foods and to understand what cholesterol and saturated fat is. Similarly, Nguyen (2007) found that the accuracy with which food items, such as apples or cookies are classified as healthy or unhealthy improves with age. In particular, she found that a sample of adults was more accurate than 7-yo, 4-yo and 3-yo children, and that 3-yo children’s accuracy significantly differed from 4-yo and 7-yo children’s accuracy. Similar results were reported by Tatlow-Golden et al. (2013) who examined 3-to5-yo children’s accuracy in the same ability and found that accuracy increases significantly from 3 to 5 years of age and especially from 4 to 5 years (Tatlow-Golden et al., 2013). The finding that understanding of healthy foods increases markedly from 4 to 5 years was replicated by Gripshover and Markman (2013). They noticed that 4- to 5-year old children who were exposed to a «rich conceptual framework that helped children understand the need to eat a variety of foods, [such as vegetables]» had a higher understanding of the variety, nutrient and digestion concepts, and biological functions of foods than their non-exposed counterparts (Gripshover and Markman, 2013, p. 1541). Vegetable consumption also increased significantly in the first group of children while remaining unchanged in the second group (Gripshover and Markman, 2013). Therefore, the targeted ability may be already present, in a limited way, in young children, which implies a certain potential for gaining an understanding of healthy foods in early ages. In addition, Varela and Salvador (2014) developed the so-called “structured sorting ballot” tool, which consists of four emoticons that correspond to the following categories: healthy food, unhealthy food, a liked food and a disliked food. The aim of this tool is to measure 5-, 7- and 9-yo children’s food-related health and hedonic perception (Varela and Salvador, 2014). The authors noticed that children of the three age cohorts were able to place foods in health- and pleasure-related categories at the same time, although the task took longer with the younger children (Varela and Salvador, 2014).

Dial and Musher-Eizenman (2019) found that the type of visual and verbal information that is used by children might influence children’s taste and health evaluations of foods, when it comes to categorize novel foods into health- and taste-related attributes. By attending to the appearance of these foods, they found that 4-to-6-yo children prefer novel packaged foods over novel fruits or vegetables and novel foods concealed in a box. Moreover, the children rated the first type of foods as “yummy” more often than in the case of the two other products, which the authors attribute to a possible association with marketing strategies. Children also manifested willingness to try the novel packaged foods more often than the other foods when they had to consider the appearance of these foods. However, there was no impact of food appearance on children’s health evaluations of those novel foods as “healthy” or “junky” and there was no effect of verbal information on children’s evaluations of foods. Dial and Musher-Eizenman (2019) also investigated the effect of provision of verbal information by pairing the food with the message: “tastes delicious”. The participating children did not indicate that a food was “yummy” more often than when this message was absent, which the authors attributed to children’s prudence regarding adult’s taste judgments. Children’s evaluations of foods as healthy, on the other hand, were significantly affected by verbal information, since the children reported that the foods were healthy more often when these foods were paired with the message “makes you strong” than when this message was absent. Children were also more willing to try the foods more often when they were paired with the verbal cue “good” than when no verbal information was provided. Finally, the frequency that a child said that a food that “makes you strong” is healthy increased with age, whereas no correlation was found between age and the frequency that a child said that a food that “tastes delicious” is yummy. However, regarding how children evaluated food healthiness and tasting using visual information, a negative correlation was found between child age and how often a child reported that fruits/vegetables were “yucky” and “junky” (Dial and Musher-Eizenman, 2019).

In light of the results of both of these sets of studies, we may conclude that children younger than 5−6-yo may show a limited or incipient understanding of the meaning of “healthy” and that young children’s understanding of healthy foods progresses between 4 and 5-yo. This suggests that the particular 4−5 years age range might be a sensitive period for learning to understand health-related abstract concepts if exposure to a “rich conceptual framework” takes place during that time. Children’s ability to categorize foods as healthy corresponds to a development-related ongoing cognitive process that may be present in an incipient way: at least between 3 and 7-yo. The type of cues children are exposed to: visual *vs* verbal information, may modulate their health and taste evaluations, and accuracy increases with age.

The results of these studies are conflicting in that the first set of studies concluded that young children do not have the ability to understand health-related concepts whereas the second set of studies concluded that young children can, but in a more limited way. This discrepancy can be explained with the suggestion that cognitive development, and not biological age, might impact young children’s understanding of abstract health-related concepts. According to this affirmation, there could be young children located at the concrete operational stage of cognitive development and older children located at the preoperational stage of cognitive development. The finding that Contento (1981) classified several children, who, in view of their age, should have been classified as preoperational stage children, into the concrete operational stage and *vice versa*, supports that possibility. Therefore, future Piagetian studies should first measure children’s stage of cognitive development by means of Piagetian tasks rather than directly assuming it from the child’s biological age.

On the other hand, the question remains whether understanding abstract concepts, such as food healthiness, and possessing nutritional knowledge actually helps children choose healthy foods, such as vegetables. Addressing this question, Tarabashkina et al. (2016) found, in a sample of 7-to-13-yo children, that 11-to-13yo children who manifested higher nutritional knowledge frequently consume more products that are less healthy, such as fast food, because of its appealing taste and social acceptability. In children younger than 11 years, the parent’s, rather than the children’s, nutritional knowledge weakened the impact of taste and social acceptability. Nguyen et al. (2015) similarly found that «taste trumps health» (Nguyen et al., 2015, p. 108) in 4- and 6-yo children and young adults, with a mean age of around 20 years, when faced with making food selections. In particular, participants’ healthiness ratings were not associated with their reported intention to consume a food whereas taste-related likings were (Nguyen et al., 2015). Participants were provided with neither a purpose nor a context for guiding their food choices in this study, so taste may have been used as the default selection criterion or may be a more informative cue than health, since the taste may inform us about its palatability and nutritional content (Nguyen et al., 2015).

Examining children’s food choices when they are provided with particular contexts, Marty et al. (2018) presented foods in pleasure and health related contexts. They found that only liking a flavor significantly predicted food choices of healthy foods, such as, red apple and banana *vs.* unhealthy foods, such as, chocolate cake and donut- in pleasure-related contexts (*i.e.*, a fictive birthday party), while both healthiness and liking a flavor significantly predicted food choices in a health-related context (*i.e.*, fictive nutrition class). Moreover, children chose healthier products in the health-related context than in the pleasure-related context. Therefore, even older children tend to use taste-related affective evaluations to make their food selection: especially if there is no health-based context, in which case children use additional health-related cognitive evaluations to make their food choices.

The study by Tatlow-Golden et al. (2014) dealt with the development of 2-to-5-yo children’s brand knowledge of healthy and unhealthy foods, which had been highly advertised on television, as a predictor of food request, purchase, and consumption. They found that brand knowledge increased from 3 to 5 years and that 4-yo children showed significantly greater brand knowledge than 3-yos. This allows the conclusion that there is an advance of this kind of knowledge between 3 and 4 years. Children’s knowledge of unhealthy food brands was found to be greater than their knowledge of healthy food brands, and their healthy food brand knowledge was not related to their television viewing, their mother’s education, or parent or child eating. Parent eating and children’s age were, however, found to be independent predictors of children’s knowledge of unhealthy brands. Therefore, children’s brand knowledge and its effects develop earlier (between 3 and 4 years) than children’s understanding of food healthfulness and its effects (between 4 and 5 years) (Tatlow-Golden et al., 2014). One of the effects of young children’s knowledge of brands offering high sugar-content products, and therefore unhealthy foods, refers to increases in their body mass index, as found by Cornwell et al. (2014) in 3-to-6-yo children. Thus, brand knowledge, in addition to an understanding of healthiness of a food, is another cognitive attribute that may impact young children’s food choices.

In light of the thoroughness of the evidence discussed on this section, we may broadly conclude that infants and children appear to use flavor as almost the only criterion for preferring and selecting the foods during infancy and early childhood. This is in contrast to adults who, according to Navarro-Allende et al. (2008), are able to contemplate the consequent health-related effects of eating a food, in addition to whether they like the flavor, when it comes to selecting an item and to reporting food preferences. Children however, deduce and understand other criteria and begin to use abstract concepts, such as food healthiness and causal health-related attributions, when they move from the preoperational to the concrete operational stage of cognitive development (Piaget, 1974). A large number of the common nutrition and food-related terms, such as high sugar, high sodium, high fat content, low fat, vitamin B12 or even taste are abstract concepts, since we cannot see these components in food (Lytle et al., 1997).

A further conclusion is that factors other than an understanding of healthiness, may impact children’s food choices in some child populations. In particular, appealing taste and social acceptability of foods may have an influence on food choices in early adolescence (Tarabashkina et al., 2016). Parents’ nutritional knowledge and children’s brand knowledge, which is another cognitive attribute of foods, may have the same effect in young children (Tarabashkina et al., 2016; Tatlow-Goden et al., 2014).

The research has shown that brand knowledge of unhealthy foods that are highly advertised on TV significantly increases between 3 and 4 years of age (Tatlow-Goden et al., 2014) and understanding abstract health-related concepts after exposure to “rich conceptual frameworks” significantly increases between 4 and 5 years of age (Gripshover and Markman, 2013). This suggests the hypotheses that these age ranges might be potential sensitive periods for acquisition of brand knowledge of healthy foods, such as vegetables and for understanding abstract concepts, such as healthiness of a given food. Confirmation of this hypothesis in the future would lead to recommendations to expose young children to brand knowledge of healthy foods, such as vegetables, from 3 years of age, and to develop educational strategies to provide health-related nutritional knowledge from 4 years of age. Both of these actions might help increase vegetable consumption by young children.

## 4 On how brain development maps cognitive development and the relationship between this process and children’s vegetable consumption

Examination and understanding of potential parallels between brain maturation of sensory systems and cognitive development may contribute to identify the nature of the overlap of these processes and their involvement in children’s vegetable consumption. Previous neuroimaging research, which has been mainly focused on food-related visual images, has found that there is a progressive increase of neural activity in high-order processing from late childhood to young adulthood in response to visually presented food-related (Kilgore and Yurgelun-Todd, 2005) and food stimuli (Van Meer et al., 2015). Indeed, neuro-activation elicited by visual stimulation may provide insights into the neural development of chemo-sensory systems since only low-order brain regions are involved at early stages of development, such as young childhood. This might also be applicable in the case that the particular stimulus is a real food and not only a picture of a food. Therefore, a reasonable hypothesis is that there is also less neural activity in high-order processing brain areas than in low-order processing brain areas in response to those kinds of stimuli at young childhood. Confirmation of this hypothesis would be in agreement with results of the research discussed above that shows that young children are either limited in their use and understanding of abstract concepts, such as healthy foods or the healthiness of a given food, or may even be incapable of using or understanding that kind of information. To either use or understand that kind of information, or to do it more accurately, would require either involvement of high-order brain areas or their involvement to a greater extent. Logical abstract thinking develops later in life (Contento, 1981) and the brain areas associated with logical and relational thinking, such as the RLPFC, require a prolonged maturational course (Dumontheil, 2014). It therefore makes sense that young children typically use food-related sensory information, such as the flavor of a given food, rather than food-related cognitive attributes, such as the healthiness of a particular food or its post-ingestive effects on his or her body. From this developmental point of view, therefore, it also makes sense that young children tend to reject vegetable consumption, since they usually do not use or understand the cognitive attributes of vegetables, such as healthiness. In terms of brain and cognitive growth, they may not be able to do it, but they are able to perceive the bad taste of vegetables.

On the other hand, the findings by Van Meer et al. (2016) suggest that older children/adolescents show a greater readiness to consume unhealthy foods than healthy foods and in a greater extent than adults do. Unhealthy foods, such as fast food and industrial baking are usually highly savory and energy-laden edibles. Neural processing of the hedonic value of chemical stimuli occurs from birth (see Rohlfs-Domínguez, 2011a, for a review) and the reward of those stimuli takes place at least from late childhood (Rohlfs-Domínguez, 2011a; English et al., 2016; Fearnbach et al., 2016; English et al., 2017). Therefore, we hypothesize that both neural processing of the hedonic value and neural processing of reward of chemical stimuli are also present at young childhood. These findings, and this hypothesis, on the brain maturation of chemical senses are in agreement with the findings of the sets of studies on cognitive development and are in agreement with the findings by Nguyen et al. (2015) who found that young children are, from a developmental perspective, able to use sensory food-related information when they are asked to make food choices or to report food preferences.

**Fig. 5:**
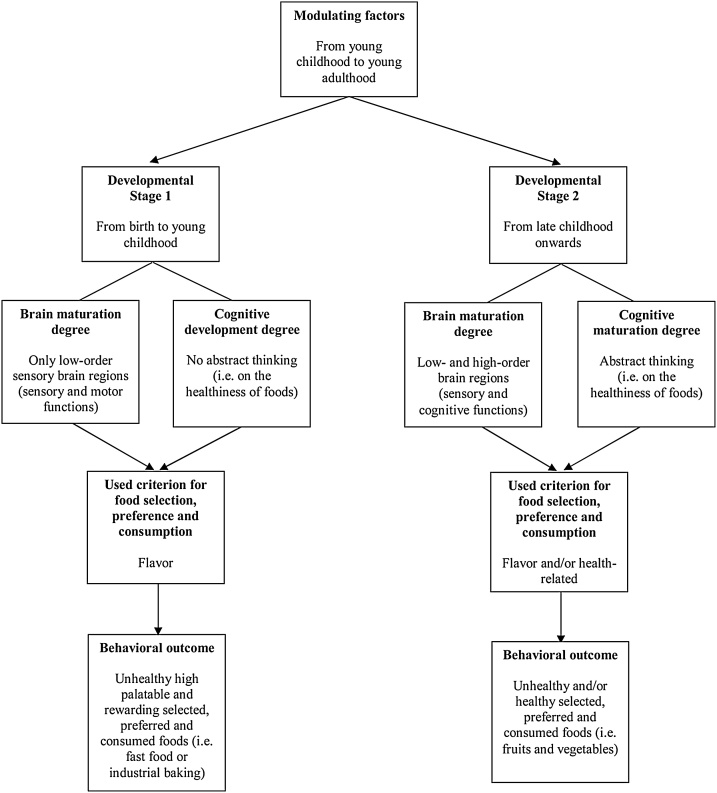
Graphical representation of the explanatory theoretical model on brain and cognitive changes that lead children to consume vegetables on a regular basis, where there are two major stages of development to observe: developmental stage 1 and developmental stage 2.

These results, combined with the Piagetian theory of development, led to the development of a theoretical model that may predict, at least partially, the development-related changes that have to occur at the brain and cognitive levels for children to make vegetable-based food choices (Fig. 5).

At the beginning of the first major developmental stage, sensory functions, such as taste and smell are already present and the underlying low-order sensory brain regions, such as subcortical areas, are functional, which means that humans are able to perceive chemical stimuli from birth. Cognitive functions, such as abstract thinking, however, are limited or even absent at the early stages of development, and the underlying brain areas, such as the frontal cortical areas, are not yet sufficiently developed to understand abstract concepts, such as healthy foods. The consequence is that health-related considerations, which are abstract concepts by nature, are not considered by young children when they make food choices so they prefer good tasting edibles, such as pizza or candies rather than healthy food items, such as vegetables. As development continues, high-order brain areas gradually reach sufficient levels of development that they become increasingly engaged in making food choices, so that individuals may take abstract issues, such as the healthiness of a food, into account. This leads to higher rates of vegetables-based food choices.

In light of the reviewed literature, a series of factors appear to modulate the nature of the graphically represented components of the model. Exposure to “rich conceptual environments” may increase the ability of 4- to 5-yo children to understand abstract health-related concepts and thus to consume vegetables (Gripshover and Markman, 2013). Furthermore, exposure to verbal cues may increase how often 4- to 6-yo young children evaluate foods, including vegetables, as healthy, with this tendency increasing with age (Dial and Musher-Eizenman, 2019). Both of these factors might be explained by the plastic nature of brain, which allows children to learn abstract concepts, which in turn might compensate for the incomplete brain and cognitive development of the early first developmental stage, and thus encourage young children to consume vegetables. Exposure to TV-advertisements of healthy foods, such as vegetables, might facilitate 3- to 5-year-old children to increase their brand knowledge of these foods (Tatlow-Golden et al., 2014), which might help them choose vegetables. The nutritional knowledge of parents helps children younger than 11-yo make vegetable-based food choices (Tarabashkina et al., 2016) although the appealing taste and social acceptability of less healthy foods leads to 11- to 13-yo children preferring them, despite of their nutritional knowledge (Tarabashkina et al., 2016). The taste of a food appears to be a more informative cue than health or lack of purpose or context in guiding food choices by 4- and 6-yo children: a phenomenon that also applies to the reported intentions to consume a given food by 20-year olds (Nguyen et al., 2015). Provision of a pleasure context can facilitate 6- to 11-year old children to make food selections on the basis of the flavor, while a health-related context can lead to them using both healthiness and flavor as decision criteria (Marty et al., 2018). Future developmental neuroimaging studies could elucidate the neural circuitry of these modulating factors, which would further our understanding of the correlational and/or causal nature of the overlap between brain maturation of chemical senses and cognitive development.

Finally, there is a series of well-known environmental factors that may also have an impact on children’s food preferences and/or choices. These factors include the social-affective context (*e.g.* Birch et al., 1980; Addessi et al., 2005), whether a food is easy to get (Michela and Contento, 1986), parents’ feeding practices (see Larsen et al., 2015; Litchford et al., 2020; Rahill et al., 2020 for comprehensive reviews), peers’ and friends’ influence (*e.g.* Salvy et al., 2012) and having the opportunity to choose foods (*e.g.* Rohlfs Domínguez et al., 2013; De Costa et al., 2017). Thorough discussion of these factors is beyond the scope of this contribution and it remains the challenge of future research to investigate these factors more thoroughly.

## 5 Final remarks

This review aimed to understand the nature of the overlap between brain maturation and cognitive development and the parallel involvement of these processes in influencing child vegetable consumption. For this purpose, and as a first step, we reviewed the currently available evidence of relevant developmental-related changes in brain activation patterns that have been obtained through neuroimaging techniques -fMRI-. Most of these studies have used visual images of food-related stimuli instead of chemical stimuli, such as odors, tastes or real foods or drinks, and future researchers are recommended to use these kinds of stimuli in future fMRI studies. As a second step, we reviewed the current state of the art regarding the relationship between children’s cognitive development and their understanding of abstract health-related terms, such as healthy foods in terms of vegetables and vegetable consumption.

The review has identified clear evidence that brain activation patterns in response to food-related stimuli, indeed, change across brain maturation and that use or understanding of abstract terms, such as the healthiness of vegetables, also changes with mental growth. Additionally, from the two-fold analysis, this review has inferred some parallels between both developmental processes: brain maturation and cognitive development, which may help understand their overlaps and their joint involvement in child vegetable consumption. Piagetian development-related theory was especially useful for making these inferences. This has led us to propose a theoretical predictive model of the development-related brain and cognitive changes that encourage children to consume vegetables regularly.

In view of the available fMRI studies, which have been reviewed here, we can conclude that almost only low-order, or sensory-motor brain regions, may be involved at early stages of development, such as young childhood, when it comes to visualizing pictures of food-related stimuli and probably real foods or drinks. Behavioral studies demonstrate that infants and young children are able to perceive tastes and odors. Such cues may play an important role in processing hedonic value and choice of foods. This further implies that young children do not use, or cannot understand, abstract concepts, such as the healthiness of vegetables, which is a tool that requires involvement of high-order brain regions that develop later. However, future fMRI studies should use real foods, such as vegetables or drinks, such as vegetable juices as stimuli in gustatory and olfactory tasks in order to further elucidate the relationship between the development of the olfaction/gustation systems in the brain and vegetable consumption.

The main contribution of this review is to enhance awareness within the scientific community of the need to conduct further developmental research on both of the targeted levels: brain and mental, in order to make progress in our understanding of how both are involved in influencing child vegetable consumption. A promising line of future research would be to examine child brain activation patterns in response to food stimuli at the 3–4 and 4−5 age ranges, which are potential sensitive periods for acquisition of brand knowledge of foods and health-related abstract concepts. Furthermore, we recommend further study to confirm the existence of these particular sensitive periods and to examine their potential impact on child vegetable consumption: thus contributing to our integral understanding of the three targeted study levels: brain, cognition and behavior.

## Statement of the individual author’s contributions

The author has done all the required work of the present contribution.

## Funding

I would like to thank to Dr. Contento for her recommendations on bibliography and to Dr. Home for his effort in reviewing this paper by making interesting suggestions on the content and refining the English language of the text of the manuscript.

## Declaration of Competing Interest

None.

## References

[bib0005] Addessi E., Galloway A.T., Visalberghi E., Birch L.L. (2005). Specific social influences on the acceptance of novel foods in 2-5 year old children. Appetite.

[bib0010] Anderson P.M., Butcher K.E. (2006). Childhood obesity: trends and potential causes. Future Child.

[bib0015] Aranceta-Batrina J., Pérez-Rodrigo C. (2016). Determinants of childhood obesity: ANIBES study. Nutr. Hosp..

[bib0020] Bahn K.D. (1989). Cognitively and perceptually based judgements in children’s Brand discriminations and preferences. J. Bus. Psychol..

[bib0025] Bailey D.B. (2002). Are critical periods critical for childhood education? The role of timing in early childhood pedagogy?. Early Child. Res. Q..

[bib0030] Baskale H., Bahar Z., Günsel B., Ari M. (2009). Use of Piaget’s theory in preschool nutrition education. Rev. Nutr..

[bib0035] Beauchamp G.K., Mennella J.A. (1998). Sensible Phasen in der Entwicklung von Geschmacksempfindungen und –vorlieben bei Menschen. Annales Nestlé.

[bib0040] Beauchamp G.K., Mennella J.A. (2011). Flavor perception in human infants: development and functional significance. Digestion.

[bib0045] Binder J.R., Fernandino L. (2015). Semantic processing. Brain Mapping.

[bib0050] Birch L.L. (1999). Development of food preferences. Annu. Rev. Nutr..

[bib0055] Birch L.L., Zimmerman S.I., Hind H. (1980). The influence of social-affective context on the formation of children’s food preferences. Child Dev..

[bib0060] Burguess-Champoux T., Marquart L., Vickers Z., Reicks M. (2006). Perceptions of children, parents, and teachers regarding whole-grain foods, and implications for a school-based intervention. J. Nutr. Educ. Behav..

[bib0065] Contento I. (1981). Children’s thinking about food and eating. A Pigatian-based study. J. Nutr. Educ..

[bib0070] Cornwell T.B., McAlister A.R., Polmear-Swendis N. (2014). Children’s knowledge of packaged and fast food brands and their BMI.WHy the relationship matters for policy makers. Appetite.

[bib0075] De Costa P., Møller P., Bom Frøst M., Olsen A. (2017). Changing children’s eating behavior. A review of experimental research. Appetite.

[bib0080] Dial L.A., Musher-Eizenman D.R. (2019). Healthy? Tasty? Children’s evaluative categorization of novel foods. Cogn. Dev..

[bib0085] Dumontheil I. (2014). Development of abstract thinking during childhood and adolescence: the role of rostrolateral prefrontal cortex. Dev. Cogn. Neurosci..

[bib0090] English L., Lasschuijt M., Keller K.L. (2015). Mechanisms of the portion size effect. What is known and where do we go from here?. Appetite.

[bib0095] English L.K., Fearnbach S.N., Lasschuijt M., Schlegel A., Anderson K., Harris S., Wilson S.J., Fisher J.O., Savage J.S., Rolls B.J., Keller K.L. (2016). Brain regions implicated in inhibitory control and appetite regulation are activated in response to food portion size and energy density in children. Int. J. Obes..

[bib0100] English L.K., Fearnbach S.N., Wilson S.J., Fisher J.O., Savage J.S., Rolls B.J., Keller K.L. (2017). Food portion size and energy density evoke different patterns of brain activation in children. Am. J. Clin. Nutr..

[bib0105] Faas A.E., Spoltón E.D., Moya P.R., Molina J.C. (2000). Differential responsiveness to alcohol odor in human neonates effects of maternal consumption during gestation. Alcohol.

[bib0110] Fallon A.E., Rozin P., Pliner P. (1984). The child’s conception of food: the development of food rejections with special reference to disgust and contamination sensitivity. Child Dev..

[bib0115] Fearnbach S.N., English L.K., Lasschuijt M., Wilson S.J., Savage J.S., Fisher J.O., Rolls B.J., Keller K.L. (2016). Brain response to images of food varying in energy density is associated with body composition in 7- to 10-year-old children: results of an exploratory study. Physiol. Behav..

[bib0120] Frerichs L., Intolubbe-Chmil L., Brittin J., Teitelbaum K., Trowbridge M., Huang T.T.-K. (2016). Children’s discourse of liked, healthy, and unhealthy foods. J. Acad. Nutr. Diet..

[bib0125] Gogtay N., Giedd J.N., Lusk L., Hayashi K.M., Greenstein D., Vaituzis A.C., Nugent T.F., Herman D.H., Clasen L.S., Toga A.W., Rapoport J.L., Thompson P.M. (2004). Dynamic mapping of human cortical development during childhood through early adulthood. Procedings of the National Academy of Sciences of the United States of America.

[bib0130] Gripshover S.J., Markman E.M. (2013). Teaching young children a theory of nutrition: conceptual change and the potential for increased vegetable consumption. Psychol. Sci..

[bib0135] Havermans R.C., Etievant P., Guichard E., Salles Ch., Voilley A. (2016). Learning of human flavor preferences. Flavor. From Food to Behaviors, Wellbeing and Health. A Volume in Woodhead Publishing Series in Food Science, Technology and Nutrition.

[bib0140] Heymann H., Ebeler S.E. (2017). Sensory and Instrumental Evaluation of Alcoholic Beverages.

[bib0145] Holsen L.M., Zarcone J.R., Thompson T.I., Brooks W.M., Anderson M.F., Ahluwalia J.S., Nollen N.L., Savage C.R. (2005). Neural mechanisms underlying food motivation in children and adolescents. NeuroImage.

[bib0150] Keller K.L., Adise S. (2016). Variation in the ability to taste bitter thiourea compounds: implications for food acceptance, dietary intake, and obesity risk in children. Annu. Rev. Nutr..

[bib0155] Keller K.L., Bruce A.S., Lumeng J., Fischer J. (2018). Neurocognitive influences on eating behavior in children. Pediatric Food Preferences and Eating Behaviors.

[bib0160] Killgore W.D.S., Yurgelun-Todd D.A. (2005). Developmental changes in the functional brain responses of adolescents to images of high and low-calorie food. Developmental Psychobioiogy.

[bib0165] Kringelbach M.L., Kirkham T.C., Cooper S.J. (2007). Cortical systems involved in appetite and food consumption. Appetite and Body Weight. Integrative Systems and the Development of Anti-Obesity Drugs.

[bib0170] Larsen J.K., Hermans R.C.J., Sleddens E.F.C., Engels R.C.M.E., Kremers S.P.J. (2015). How parental dietary *behavior* and *food* parenting practices affect children's dietary *behavior*. Interacting sources of influence?. Appetite.

[bib0175] Leahy K.E., Birch L.L., Fisher J.O., Rolls B.J. (2008). Reductions in entrée energy density increase children’s vegetable intake and reduce energy intake. Obesity (Silver Spring).

[bib0180] Litchford A., Roskos M.R.S., Wengreen H. (2020). Influence of fathers on the feeding practices and behaviors of children: a systematic review. Appetite.

[bib0185] Lourenco F., Casey B.C. (2013). Adjusting Behavior to changing environmental demands with development. Neurosci. Behavav. Rev..

[bib0190] Lytle L.A., Eldridge A.L., Kotz K., Piper J., Williams S., Kalina B. (1997). Children’s interpretation of nutrition messages. J. Nutr. Educ. Behav..

[bib0195] Marty L., Nicklaus S., Miguet M., Chambaron S. (2018). When do healthiness and liking drive children’s food choices? The influence of social context and weight status. Appetite.

[bib0200] McNeal J.U. (2007). On Becoming a Consumer. The Development of Consumer Behavior Patterns in Childhood..

[bib0205] Medler K. (2008). Signaling mechanisms controlling taste cell function. Critical Rev. Eukariot Gene Expres..

[bib0210] Mennella J.A., Pepino M.Y., Reed D.R. (2005). Genetic and environmental determinants of bitter perception and sweet preferences. Pediatrics.

[bib0215] Michela J.L., Contento I.R. (1984). Spontaneous classification of foods by elementary school aged children. Health Educ. Q..

[bib0220] Michela J.L., Contento I.R. (1986). Cognitive, motivational, social and environmental influences on children’s food choices. Health Psychol..

[bib0225] Mukherjee P., McKinstry R.C. (2006). Diffusion tensor imaging and tractography of human brain development. Neuroimaging Clin. N. Am..

[bib0230] Navarro-Allende A., Khataan N., El-Sohemy A. (2008). Impact of genetic and environmental determinants of taste with food preferences in older adults. J. Nutr. Elder..

[bib0235] Nguyen S.P. (2007). An apple a day keeps the doctor away: children’s evaluative categories of food. Appetite.

[bib0240] Nguyen S.P., Girgis H., Robinson J. (2015). Predictors of children’s food selection: the role of children’s perceptions of the health and taste of foods. Food Qual. Prefer..

[bib0245] Nicklaus S., Boggoi V., Chabanet C., Issanchou S. (2005). A prospective study of food seeking in childhood, adolescence and early adult life. Appetite.

[bib0250] Nu Ch.T., MacLeod P., Barthelemy J. (1996). Effects of age and gender on adolescents’ food habits and preferences. Food Qual. Prefer..

[bib0255] Pearson N., Biddle S.J., Gorely T. (2009). Family correlates of breakfast consumption among children and adolescents. Appetite.

[bib0260] Pérez-Rodrigo C., Ribas L., Serra-Majem L.I., Aranceta J. (2003). Food preferences of Spanish children and young people: the enKid study. Eur. J. Clin. Nutr..

[bib0265] Piaget J. (1974). Understanding Causality.

[bib0270] Pulaski M.A.S. (1997). El desarrollo de la mente infantil según Piaget.

[bib0275] Qi Y., Niu J. (2015). Does childhood nutrition predict health outcomes during adulthood? Evidence from a population-based study in China. J. Biosoc. Sci..

[bib0280] Rahill S., Kennedy A., Kearny J. (2020). A review of the influence of fathers on children’s eating behaviours and dietary intake. Appetite.

[bib0285] Rohlfs Domínguez P., Gámiz F., Gil M., Moreno H., Márquez Zamora R., Gallo M., de Brugada I. (2013). Providing choice increases children’s vegetable intake. Food Qual. Prefer..

[bib0290] Rohlfs-Domínguez P. (2011). The study of postnatal and later development of taste and olfactory systems using the human brain mapping approach: an update. Brain Res. Bull..

[bib0295] Rohlfs-Domínguez P. (2011). Flavor exposure during sensitive periods of development as a key mechanism of flavor learning: implications for future research. Am. J. Clin. Nutr..

[bib0300] Rohlfs-Domínguez P. (2014). Development and acquisition of flavor and food preferences in children: an update until 2010. J. Food Res..

[bib0305] Rohlfs-Domínguez P. (2014). Promoting our understanding of neural plasticity by exploring developmental plasticity in early and adult life. Brain Res. Bull..

[bib0310] Rohlfs-Domínguez P. (2017). Insights into the impact of genetic variation in bitter taste sensitivity on young children’s acceptance of vegetables and body mass index: an update until 2010. Integr. Food Nutr. Metab..

[bib0315] Rohlfs-Domínguez P. (2018). A minireview of effects of maternal diet during pregnancy on postnatal vegetable consumption: Implications for future research (a new hypothesis) and recommendations. Crit. Rev. Food Sci. Nutr..

[bib0320] Rolland-Cachera M.F., Akrout M., Péneau S. (2016). Nutrient intakes in early life and risk of obesity. Int. J. Environ. Res. Public Health.

[bib0325] Rosenstein D., Oster H. (1988). Differential facial responses to four tastes in newborns. Child Dev..

[bib0330] Salvy S.J., de la Haye K., Bowker J.C., Hermans R.C.J. (2012). Influence of peers and friends on children’s and adolescents’ eating and activity behaviors. Physiol. Behav..

[bib0335] Simpson K., Sweazey R., Hanes D. (2006). Olfaction and taste. Fundamental Neuroscience for Basic and Clinical Applications.

[bib0340] Spahn J.M., Callahan E.H., Spill M.K., Wong Y.P., Benjamin-Neelson S.E., Birch L., Black M.M., Cook J.T., Faith M.S., Mennella J.A., Casavale K.O. (2019). Influence of maternal diet on flavor transfer to amniotic fluid and breast milk and children’s responses: a systematic review. Am. J. Clin. Nutr..

[bib0345] Spill M.K., Johns K., Callahan E.H., Shapiro M.J., Wong Y.P., Benjamin-Neelson S.E., Birch L., Black M.M., Cook J.T., Faith M.S., Mennella J.A., Casavale K.O. (2019). Repeated exposure to food and food acceptability in infants and toddlers: a systematic review. Am. J. Clin. Nutr..

[bib0350] Tarabashkina L., Quester P., Crouch R. (2016). Exploring the moderating effect of children’s nutritional knowledge on the relationship between product evaluations and food choice. Soc. Sci. Med..

[bib0355] Tatlow-Golden M., Hennessy E., Dean M., Hollywood L. (2013). ‘Big, strong and healthy’. Young children’s identification of food and drink that contribute to healthy growth. Appetite.

[bib0360] Tatlow-Golden M., Hennessy E., Dean M., Hollywood L. (2014). Young children’s food brand knowledge. Early development and associations with television viewing and parent’s diet. Appetite.

[bib0365] Toccio S., Kline-Fath B., Kanal E., Schmithorst V.J., Panigray A. (2015). MRI evaluation and safety in the developing brain. Semin. Perinatol..

[bib0370] Van Duyn M.A.S., Pivonka E. (2000). Overview of the health benefits of fruit and vegetable consumption for the dietetics professional: selected literature. J. Am. Diet. Assoc..

[bib0375] Van Meer F., van der Laan L.N., Adan R.A.H., Viergever M.A., Smeets P.A.M. (2015). What you see is what you eat: an ALE meta-analysis of the neural correlates of food viewing in children and adolescents. NeuroImage.

[bib0380] Van Meer F., van der Laan L.N., Charbonnier L., Viergever M.A., Adan R.A., Smeets P.A. (2016). Developmental differences in the brain response to unhealthy food cues: an fMRI study of children and adults. Am. J. Clin. Nutr..

[bib0385] Varela P., Salvador A. (2014). Structured sorting using pictures as a way to study nutritional and hedonic perception in children. Food Qual. Prefer..

[bib0390] Wadhera D., Capaldi Phillips E.D., Wilkie L.M. (2015). Teaching children to like and eat vegetables. Appetite.

[bib0395] Warren E., Parry O., Lynch R., Murphy S. (2008). If I don’t like it then I can choose what I want’: welsh school children’s accounts of preference for and control over food choice. Health Promot. Int..

[bib0400] Yoshida R., Yasumatsu K., Shigemura N., Ninomiya Y. (2006). Coding Channels for taste perception: information transmission from taste cells to gustatory nerve fibers. Arch. Histol. Cytol..

[bib0405] Yzydorczyk C., Mitanchez D., Boubred F., Simeone U., Watsin R.R., Dokken B.B. (2015). Early origins of health and disease. Glucose Intake and Utilization in Pre-Diabetes and Diabetes, Part I.

[bib0410] Zalesky A., Pantelis C., Cropley V., Fornito A., Cocchi L., McAdams H., Clasen L., Greenstein D., Rapoport J.L., Gogtay N. (2015). Delayed development of brain connectivity in adolescents with schizophrenia and their unaffected siblings. Journal of the American Medical Association Psychiatry.

[bib0415] Zeinstra G.G., Koelen M.A., Kok F.J., de Graaf C. (2007). Cognitive development and children’s perceptions of fruit and vegetables; a qualitative study. Int. J. Behav. Nutr. Phys. Act..

